# Real-Time Station Grouping under Dynamic Traffic for IEEE 802.11ah

**DOI:** 10.3390/s17071559

**Published:** 2017-07-04

**Authors:** Le Tian, Evgeny Khorov, Steven Latré, Jeroen Famaey

**Affiliations:** 1 IDLab, Department of Mathematics and Computer Science, University of Antwerp—imec, 2020 Antwerp, Belgium; steven.latre@uantwerpen.be (S.L.); Jeroen.Famaey@uantwerpen.be (J.F.); 2 Network Protocols Research Lab, Institute for Information Transmission Problems, Russian Academy of Sciences, 127051 Moscow, Russia; khorov@frtk.ru

**Keywords:** IEEE 802.11ah, dense IoT networks, restricted access window (RAW), real-time RAW optimization, dynamic traffic

## Abstract

IEEE 802.11ah, marketed as Wi-Fi HaLow, extends Wi-Fi to the sub-1 GHz spectrum. Through a number of physical layer (PHY) and media access control (MAC) optimizations, it aims to bring greatly increased range, energy-efficiency, and scalability. This makes 802.11ah the perfect candidate for providing connectivity to Internet of Things (IoT) devices. One of these new features, referred to as the Restricted Access Window (RAW), focuses on improving scalability in highly dense deployments. RAW divides stations into groups and reduces contention and collisions by only allowing channel access to one group at a time. However, the standard does not dictate how to determine the optimal RAW grouping parameters. The optimal parameters depend on the current network conditions, and it has been shown that incorrect configuration severely impacts throughput, latency and energy efficiency. In this paper, we propose a traffic-adaptive RAW optimization algorithm (TAROA) to adapt the RAW parameters in real time based on the current traffic conditions, optimized for sensor networks in which each sensor transmits packets with a certain (predictable) frequency and may change the transmission frequency over time. The TAROA algorithm is executed at each target beacon transmission time (TBTT), and it first estimates the packet transmission interval of each station only based on packet transmission information obtained by access point (AP) during the last beacon interval. Then, TAROA determines the RAW parameters and assigns stations to RAW slots based on this estimated transmission frequency. The simulation results show that, compared to enhanced distributed channel access/distributed coordination function (EDCA/DCF), the TAROA algorithm can highly improve the performance of IEEE 802.11ah dense networks in terms of throughput, especially when hidden nodes exist, although it does not always achieve better latency performance. This paper contributes with a practical approach to optimizing RAW grouping under dynamic traffic in real time, which is a major leap towards applying RAW mechanism in real-life IoT networks.

## 1. Introduction

The Internet of Things (IoT) aims to provide connectivity among a huge number of “things” anytime and anywhere. This will highly impact every aspect of the world we live in, including economics, politics, and social life. Emerging IoT applications and services, such as smart meters, environmental/agricultural monitoring and automation of industrial processes, require myriads of battery-powered smart things (e.g., sensors, actuators, controllers) connected together in an energy efficient way. Currently, there are two categories of low-power IoT communication technologies: wireless personal area network (WPAN) and low-power wide area network (LPWAN) technologies. Within short transmission range (i.e., tens of meters), WPAN technologies (e.g., Bluetooth Low Energy, ZigBee) provide relatively high throughput (i.e., up to a few hundred kilobits per second), while long-range communications (i.e., up to several kilometers) can be supported by LPWAN technologies (e.g., LoRA, SigFox) at much lower throughput (i.e., up to at most a few kilobits per second). As the transmission range of the WPAN technologies is too short and throughput of the LPWAN technologies is too low, both of them can only be applied in a limited set of IoT scenarios. Therefore, a gap still exists for a low-power IoT communication technology that offers sufficient throughput over longer ranges.

The recently released IEEE 802.11ah Wi-Fi standard, marketed as Wi-Fi HaLow, fills this gap. It operates in the unlicensed sub-1 GHz frequency bands (e.g., 863–868 MHz in Europe, 755–787 MHz in China and 902–928 MHz in North-America). Similar to previous Wi-Fi standards, it supports several modulation and coding schemes (MCS) in order to offer a trade-off between throughput, range and energy efficiency. This allows it to support transmission ranges from 100 m up to 1 km with data rates between 0.15 Mbps and 346.67 Mbps. Thus, IEEE 802.11ah has the potential to achieve much higher transmission ranges than existing WPAN and much higher throughput than both WPAN and LPWAN technologies. On the MAC (Media Access Control) layer, in order to increase efficiency in the face of a large number of densely deployed, energy constrained stations, several innovative mechanisms are introduced, including hierarchical organization, short MAC header, restricted access window (RAW), traffic indication map (TIM) segmentation and target wake time (TWT).

The RAW feature aims to increase scalability in dense IoT networks, where a large number of stations connect to a single access point (AP). With RAW, stations are divided into groups, limiting simultaneous channel access to one group. Therefore, the collision probability for upstream traffic is highly reduced. However, the grouping strategy, which decides how to split stations among groups, is not mentioned in the standard. Concretely, a station grouping algorithm should be implemented at the AP side to determine the number of RAW groups, the duration of each group, and how to divide stations among them. Furthermore, these parameters should be dynamically adapted by the AP between consecutive beacon intervals. In previous work, we conducted an in-depth analysis of the influence of network and traffic conditions on the optimal station grouping parameters [1]. We concluded that the optimal parameters depend on a wide range of network variables, such as number of stations, network load and traffic patterns. This shows the need for dynamic station grouping algorithms that determine the optimal station grouping parameters based on the current network and traffic conditions. As these conditions change, the algorithm should similarly adapt.

In this article, we present a novel real-time station grouping algorithm that adapts the RAW parameters based on the current (estimated) traffic conditions, optimized for sensor networks with mainly upstream traffic. It improves upon the state of the art in three ways. First, it is designed for dynamic and heterogeneous traffic conditions, where each station has a different packet transmission interval that may change over time. Second, it only uses information readily available on the AP side, estimating station-side variables based on available data. Third, it can be executed in real time, not relying on at-runtime evaluation of complex mathematical models. The combination of these three factors allows the algorithm to be deployed in realistic environments, executing it at the start of each beacon interval for instantaneous adaptation to changes in traffic conditions. To the best of our knowledge, this is the first real-time IEEE 802.11ah station grouping algorithm that can cope with dynamic and heterogeneous traffic. The algorithm is thoroughly evaluated using our previously presented 802.11ah ns-3 simulator [2].

The remainder of this article is structured as follows. Section 2 introduces related research on IEEE 802.11ah station grouping. Section 3 provides a brief overview of the IEEE 802.11ah RAW feature. The real-time station grouping algorithm for dynamic traffic is presented in Section 4. Section 5 describes the derivation of the optimal value of two input parameters used by the proposed algorithm. In Section 6, we provide a comparison of the algorithm to enhanced distributed channel access/distributed coordination function (EDCA/DCF) and static grouping using simulation results. Finally, conclusions and future work are discussed in Section 7.

## 2. Related Work

Even though the IEEE 802.11ah standard was only officially published recently, research on IEEE 802.11ah has been conducted for several years. Several articles [3,4,5,6,7,8] provide a deep overview of the key features of the new technology, fully describing the advantages and challenges in the design of PHY and MAC layer schemes. Moreover, performance assessment on IEEE 802.11ah in four common machine-to-machine (M2M) scenarios, i.e., smart metering, agriculture monitoring, animal monitoring, and industrial automation, has been conducted by Adame et al. [5]. Baños et al. [8] thoroughly evaluated performance of IEEE 802.11ah in comparison to the most notable IEEE 802.11 standards, and exposed the capabilities of IEEE 802.11ah in supporting different IoT applications.

Several recent works study physical layer aspects of 802.11ah and sub-1 GHz communications [9,10,11,12,13,14]. Hazmi [9] conducts assessment on the link budget, and derives the achievable data rate and optimal packet size of 802.11ah. Aust and Ito [10] study three urban propagation path loss models of 802.11ah for carrier frequencies at 900 MHz. Li and Wang [14] compare the indoor coverage and time delay performance between IEEE 802.11g and IEEE 802.11ah in M2M communications. Aust and Prasad [12] proposed an IEEE 802.11ah prototype that is configured as a self-contained M2M wireless sensor system and allows an over-the-air protocol performance assessment. Casas and Papaparaskeva [13] introduced a design for a programmable 802.11ah station based on the Cadence-Tensilica Fusion digital signal processor (DSP). Aust, Prasad and Niemegeers [11] built a real-time Multiple-input, multiple-output orthogonal frequency-division multiplexing (MIMO-OFDM) testing platform for evaluating narrow-band sub-1GHz transmission characteristics. Moreover, Ba et al. [15] developed an 802.11ah fully-digital polar transmitter, this hardware prototype passes all the PHY requirements of the mandatory modes in IEEE 802.11ah with 4.4% error-vector-magnitude (EVM), while consuming only 7.1 mW with 0 dBm output power.

More relevant to the research presented in this article is work focusing on RAW analysis and optimization. Several studies have been conducted on the optimality of RAW configurations given specific network and traffic conditions [1,16,17]. Park [16] showed the effectiveness of RAW to mitigate the hidden node problem. Zhao et al. [17] evaluated RAW in terms of energy efficiency, showing that increasing the number of RAW groups significantly improves energy efficiency for sensor stations. Finally, in our own previous work [1], we evaluated the optimal RAW station grouping configuration under a variety of traffic conditions, proving the need for a dynamic regrouping algorithm, that takes into account changes in network and traffic conditions.

Several such station grouping algorithms have been proposed in literature, as shown in Table 1. The goal of these algorithms is to determine RAW parameters (i.e., number of groups and slots, group duration, and station partitioning among groups), given the current network conditions (e.g., number of stations, traffic demand, station location). For each algorithm, Table 1 shows the assumed upstream traffic conditions, the optimization objective, and the used algorithmic method. The surveyed algorithms focus on upstream traffic, as station grouping mainly improves upstream scalability, and it is the main type of traffic in sensor networks. The traffic conditions consist of two parts. First, the traffic intensity can be categorized as either (i) one packet per station per slot; (ii) saturated traffic for each station; (iii) a static finite number of packets per station per slot and (iv) a dynamic (i.e., changing from slot to slot) finite number of packets per station per slot. Second, the inter-station traffic differentiation is either homogeneous (i.e., all stations have the same traffic intensity) or heterogeneous (i.e., different stations may have different traffic intensities). The considered objectives are contention minimization (i.e., through hidden node mitigation), throughput maximization, energy consumption minimization, or a combination. It is clear that, to be applicable to real scenarios, supported traffic conditions should be both dynamic and heterogeneous.

The algorithms presented in the table are broadly based on two approaches: (i) analytical modeling and (ii) set partitioning. The analytical models make use of different techniques, such as probability theory [22,27,28], Markov chains [23,29,30], multi-objective game theory [25], and maximum likelihood estimation [26]. They all aim to optimize throughput, energy consumption, or a combination of both. Generally, they are computationally hard. This makes it infeasible to execute them in real time on actual AP hardware, where, at most, a few milliseconds are available at the start of the beacon interval to calculate a new RAW configuration. Moreover, such models require information about the station’s traffic demand that is not readily available on the AP side. To simplify the modeling process, all existing models for RAW focus on homogeneous traffic with either one packet per station or under saturation. The combination of these factors make such models useful only from a theoretical point of view, in order to analyze the effectiveness of RAW under a variety of conditions. However, they cannot be used for real-time station grouping under dynamic and realistic traffic conditions.

The set partitioning algorithms are much simpler. They assume the number of RAW slots and groups is given, and decide how to partition the associated stations among them, according to some metric. Their simplicity makes it computationally feasible to deploy them in real networks. Older work assumes simplified saturated and homogeneous network conditions. Moreover, it focuses on simple partitioning metrics, such as fully random [31] or based on the back-off timer value [24], which, in reality, is not known to the AP. Recently, some more interesting approaches have popped up. Several algorithms focus on mitigating hidden node collisions by splitting mutually hidden nodes into orthogonal groups [18,19,20]. Additionally, Chang et al. proposed a set partitioning algorithm that assumes the (static) traffic demand of each station is known by the AP and load balances them across groups [21]. Most of these recent algorithms still assume simplified homogeneous traffic [18,20]. However, two algorithms [19,21] focus on more realistic traffic, where stations have heterogeneous, static and non-saturated traffic.

Although the algorithms of Dong [19] and Chang [21] take a step in the right direction by supporting more realistic traffic and real-time execution, they still have several shortcomings that we aim to address in this article. First, none of the presented algorithms take into account traffic dynamics. In a real network, the upstream traffic intensity of stations may change over time for a variety of reasons, and the algorithm should therefore adapt to these changes. Second, they expect all information, such as the exact traffic intensity of each station, to be readily available on the AP side, which, in reality, is not the case. Third, they assume that the number of groups and slots as well as their duration are given, and only the partitioning of stations among them needs to be solved. The number of groups and their duration, however, significantly influence RAW optimality [1]. All parameters should therefore be jointly optimized.

In this paper, we present a station grouping algorithm for the IEEE 802.11ah RAW mechanism that addresses these shortcomings. It supports both dynamic and heterogeneous traffic, real-time execution, estimation of station traffic intensity on the AP side , and optimization of all RAW parameters (i.e., number of groups and slots, group duration, and station partitioning). Last but not least, it is evaluated using our IEEE 802.11ah ns-3 packet-based network simulator [2], which exhibits realistic propagation behavior (e.g., channel errors, capture effect). These propagation effects are often ignored, but significantly affect performance results.

## 3. IEEE 802.11ah Restricted Access Window

IEEE 802.11ah operates over a set of unlicensed radio bands (all in the sub-1 GHz spectrum), supporting up to 1 km transmission range, and allowing up to 8192 stations to associate with a single AP. Due to these features, 802.11ah is a highly attractive wireless communication technology for long-distance IoT use cases, such as smart meters and environmental/agricultural monitoring. This section provides an overview of the RAW station grouping feature of the 802.11ah standard, which is the focus of this article. More detailed overview of the complete standard can be found in existing literature [4,5,6,7].

**Figure 1:**
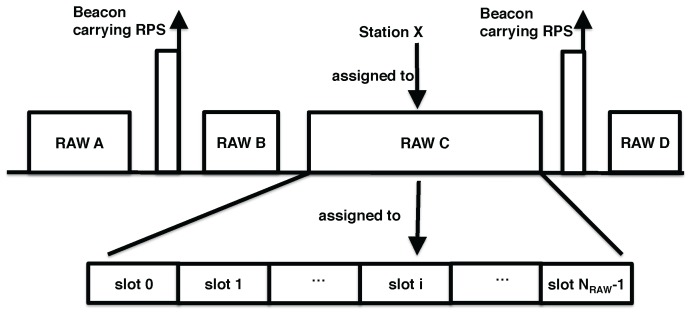
Schematic representation of the RAW mechanism, with the beacon RPS element carrying information about the number of RAW groups, their duration, number of equal-sized slots and assigned stations.

The goal of the RAW mechanism is to mitigate collisions and improve performance in dense IoT networks where a large number of stations are contending for channel access simultaneously. It splits stations into groups and only allows stations assigned to a certain group to access the channel at specific times. Figure 1 schematically depicts how RAW works. Specifically, the channel time is split into several intervals, some of which are assigned to RAW groups, while the others are considered as shared channel airtime and can be accessed by all stations. Each interval assigned to a RAW group is preceded by a beacon frame carrying a RAW parameter set (RPS) information element that specifies the RAW related information, such as the stations belonging to the group, as well as the group start time. Moreover, each RAW group consists of one or more slots, over which the stations assigned to the RAW group are evenly split (using round robin assignment). The RPS information element also contains the number of slots, slot format and slot duration count sub-fields, which jointly determine the RAW slot duration as follows [32]:

(1) D=500μs+C×120μs,

where *C* represents *slot duration count* sub-field, which is either y=11 or y=8 bits long if the slot format sub-field is set to respectively 1 or 0. The *number of slots* field is 14−y bits long. When y=11, each RAW consists of at most eight slots and the maximum value of *C* is $2^{11}-1=2047$, the slot duration is up to 246.14 ms. Otherwise, each RAW consists of at most 64 slots and the maximum value of *C* is $2^{8}-1=255$, the slot duration is limited to 31.1 ms. Stations are mapped to slots as follows [32]:

(2) $$i_{\mathrm{slot}}=\left(x+N_{\mathrm{offset}}\right)\;mod\;N_{\mathrm{RAW}},$$

where $i_{\mathrm{slot}}$ is the index of $i^{th}$ RAW slot to which the station is mapped. $N_{\mathrm{RAW}}$ is the number of slots in one RAW. $N_{\mathrm{offset}}$ is the offset value in the mapping function to improve fairness and equals the two least significant octets of the *FCS field* of the S1G beacon frame, and *x* is the index of the station. Figure 1 shows an example of the RAW slot assignment procedure.

The RPS also contains the *cross slot boundary* (CSB) sub-field. Stations are allowed to continue their ongoing transmissions even after the end of the current RAW slot when CSB is set to true. Otherwise, stations should not start a transmission if the remaining time in the current RAW slot is not enough to complete it.

**Figure 2:**
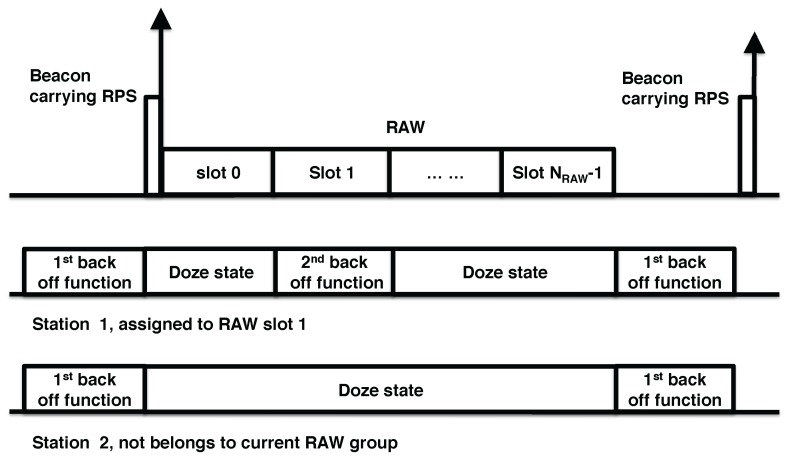
Illustration of the novel dual back-off procedure of IEEE 802.11ah.

Different from previous IEEE 802.11 standards, each station uses two back-off states of enhanced distributed channel access (EDCA) to manage transmission inside and outside their assigned RAW slot respectively (cf. Figure 2). The first back-off function state is used outside RAW slots, while the second is used inside. For the first back-off state, the station suspends its back-off timer at the start of each RAW, and restores and resumes the back-off timer at the end of the RAW. For the second back-off state, stations start back-off with initial back-off state inside their own RAW slot, and discard the back-off state at the end of their RAW slot, effectively restarting their back-off at the start of their next RAW period. As shown in Figure 2, station 1 is inside the RAW group and assigned to slot 1, while station 2 is not included in this RAW group. Therefore, station 1 uses the first back-off state outside its RAW slot period and the second back-off state inside its RAW slot, while station 2 only uses the first back-off state outside the RAW group period and goes into a sleep state inside the RAW group period.

## 4. Real-Time RAW Parameter Optimization

This section introduces the RAW optimization problem addressed in this article, and subsequently proposes the Traffic-Adaptive RAW Optimization Algorithm (TAROA). TAROA solves the RAW optimization problem in real-time, and is able to instantaneously adapt to changes in station association and traffic demand. Table 2 provides an overview of the variables used in the description of the RAW optimization problem and TAROA.

### 4.1. Problem Statement

We consider IoT sensor-based monitoring scenarios, where a large set of sensors S send measurements to a back-end server (through the AP) at specific time intervals. A sensor s∈S, also referred to as a station, has a packet transmission interval ${\hat{t}}_{int}^{s}$, which may change over time (e.g., when an environmental event triggers a change in the sensor measurement interval).

The goal of RAW optimization is to assign stations to a set of RAW slots $R^{b}$ during each beacon interval *b*, maximizing the number of successful transmissions. As an input, it uses only information readily available at the access point. The first input is the last-in-first-out (LIFO) queue of times a packet was successfully received by the AP from station *s* or $t_{\mathrm{succ}}^{s}$, where $t_{\mathrm{succ}}^{s}\left[0\right]$ represents the time of the last successful packet reception. The second input is the LIFO queue of transmission results (i.e., success or failure) $\pi_{\mathrm{trans}}^{s}$, where $\pi_{\mathrm{trans}}^{s}\left[0\right]$ is the result of the last transmission. A packet transmission is considered a failure if a RAW slot was assigned to a station, but no packet was received during that slot. The output of RAW optimization is, for each beacon interval *b*, the set of RAW slots $R^{b}$, the set of stations $S^{r}$ assigned to each slot *r*, and the duration $t^{r}$ of each slot *r*. The goal can then be formally defined as:

(3) $$\max\sum_{b}\sum_{r\in R^{b}}\sum_{s\in S^{r}}\pi_{b,r}^{s}$$

where $\pi_{b,r}^{s}$ represents the number of packets successfully received by the AP from station *s* during beacon interval *b*.

### 4.2. Traffic-Adaptive RAW Optimization Algorithm (TAROA) Overview

TAROA aims to solve the aforementioned problem by estimating the transmission interval ${\hat{t}}_{\mathrm{int}}^{s}$ of each station *s* on the AP side (as it is not known by the AP). This estimate $t_{\mathrm{int}}^{s}$ is used to determine for each beacon interval *b* the set of RAW groups and slots $R^{b}$, their duration $t^{b}$, and the stations assigned to each of them $S^{b}$.

**Figure 3:**
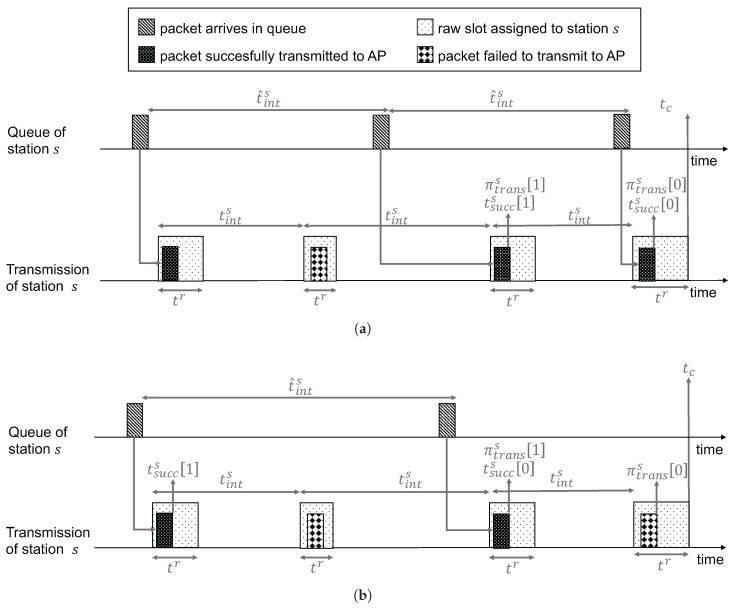
Parameters used for estimating the transmission interval of station *s* at time $t_{c}$. (**a**) transmission succeeds at time $t_{c}$; (**b**) transmission fails at time $t_{c}$.

Figure 3 depicts an example time line up to the current time $t_{c}$ for a single station attempting to transmit several packets. Station *s* places a packet in its transmit queue every ${\hat{t}}_{\mathrm{int}}^{s}$ seconds, while the access point estimates the related transmission interval as $t_{\mathrm{int}}^{s}$. The closer this estimate is to the real value, the lower the transmission latency will be for station *s*. Based on this estimate, the AP assigns RAW slots of varying duration $t^{r}$ to *s*. At the top, Figure 3a depicts an example where the last transmission attempt at time $t_{c}$ is successful. At the bottom, Figure 3b shows a related example where the last transmission attempt fails, due to a lack of packets in the queue of station *s*. For both cases, the figures graphically depict the parameter values for the last two transmissions results (i.e., $\pi_{\mathrm{trans}}^{s}\left[0\right]$ and $\pi_{\mathrm{trans}}^{s}\left[1\right]$) and the last two successful transmission times (i.e., $t_{\mathrm{succ}}^{s}\left[0\right]$ and $t_{\mathrm{succ}}^{s}\left[1\right]$). These values are used by the transmission interval estimation algorithm (i.e., Algorithm 1) to calculate $t_{\mathrm{int}}^{s}$ at the start of each beacon interval.

**Algorithm 1:** Estimate transmission interval of station s∈S
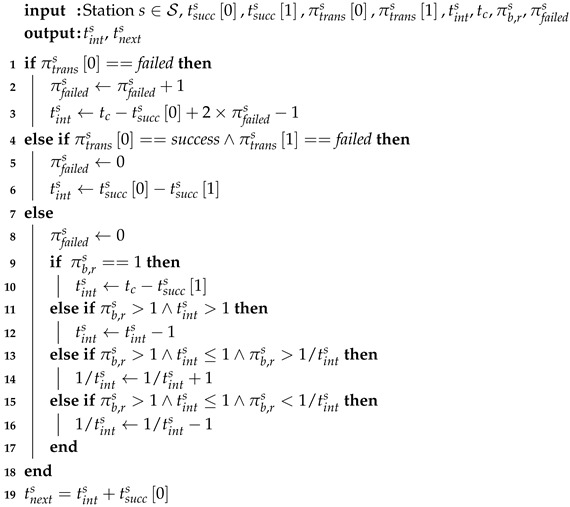


**Figure 4:**
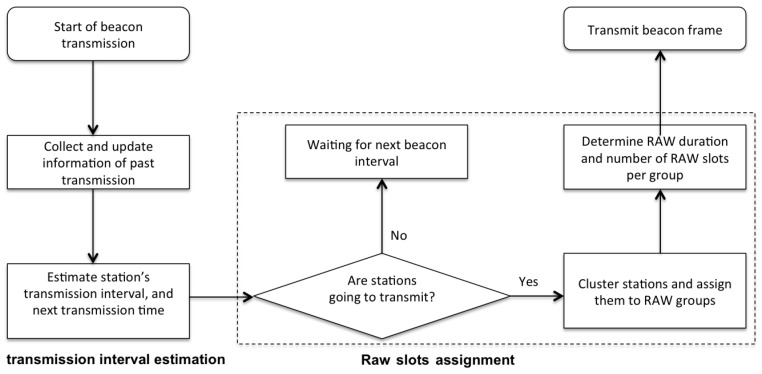
Different steps involved in the execution of the TAROA algorithm.

As depicted in Figure 4, the algorithm is executed at each target beacon transmission time (TBTT), and consists of two main steps. First, the AP updates its estimation $t_{\mathrm{int}}^{s}$ of the packet transmission interval ${\hat{t}}_{\mathrm{int}}^{s}$ of each station based on packet transmission information obtained during the last beacon interval. As the algorithm is optimized for sensor stations, it is assumed that each station transmits packets with a certain (predictable) frequency. However, as the estimation is updated at the start of each beacon interval, the algorithm can easily cope with changes in this transmission frequency over time. Second, TAROA determines the RAW parameters and assigns stations to RAW slots based on this estimated transmission frequency. Finally, the RAW parameters are transmitted to the stations by the AP in the RPS element of the beacon frame. The remainder of this section explains the two main steps of the algorithm (i.e., transmission interval estimation and RAW slot assignment).

### 4.3. Transmission Interval Estimation 

The first main step of the algorithm is determining the estimated transmission interval $t_{\mathrm{int}}^{s}$ and next transmission time $t_{\mathrm{next}}^{s}$ for each station s∈S. As shown in Algorithm 1, they are estimated based on successful and failed transmissions during the previous beacon interval. A station’s transmission is regarded as successful if the AP received at least one packet from the station. If a station was assigned a RAW slot, but no packets were received by the AP during that slot, it is considered a failed transmission. The failed transmission can be be caused by the lack of packets in the station’s transmission queue, or by a collision or interference. The algorithm consists of three main blocks: (i) the previous transmission failed (lines 1–3); (ii) the previous transmission was successful, but the one before failed (lines 4–6); and (iii) the last two transmissions were successful (lines 7–16).

First, if the previous transmission failed, the transmission failure counter $\pi_{\mathrm{failed}}^{s}$ is increased by 1 (line 2). Additionally, this means that the estimated transmission interval $t_{\mathrm{int}}^{s}$ of station *s*, is too short, as we assume *s* had no packets in its transmit queue. In reality, a transmission failure can also be caused by collisions. However, as RAW aims to minimize collisions, we can assume the probability of transmission failure caused by collision is low enough to be ignored. Increasing $t_{int}^{s}$ sharply could result in overestimation, which results in fewer packets being delivered to the AP and more packets being dropped due to the overflow of station’s transmission queue. Although increasing $t_{int}^{s}$ slowly can lead to a more accurate estimation, it may waste channel access time, as some stations will be assigned RAW slots without transmitting packets, while there will be no channel access time left for other stations that need it. For this reason, the increase of the estimated transmission interval $t_{int}^{s}$ follows the multiplicative-decrease principle of the Transmission Control Protocol (TCP) congestion control, and it is increased by the number of subsequent failed transmissions multiplied by two. As the number of failed transmission attempts increases, the algorithm assumes its estimation is more wrong and it will increase the interval faster.

In the second case (lines 4–6), the failure counter $\pi_{\mathrm{failed}}^{s}$ is set to 0 and the transmission interval is estimated as the time difference between the last two successful transmissions.

If an accurate $t_{int}^{s}$ is obtained in case 2, the next transmission will succeed and lead to case 3 (lines 7–16) in which the two last transmissions are successful. If only one packet is received (case 3.1), $t_{int}^{s}$ is updated in the same way as in case 2 (lines 9–10). If, on the other hand, $t_{int}^{s}$ is underestimated, *s* may transmit multiple packets to the AP (case 3.2, lines 11–12). In that case, the estimated transmission interval is reduced by 1 beacon interval (line 12). Finally, there are two more cases where the current estimated transmission interval is not larger than the beacon interval (i.e., $t_{\mathrm{int}}^{s}\leq 1$) and multiple packets were received from the station *s* by the AP (i.e., $\pi_{b,r}^{s}>1$ (cases 3.3 and 3.4). In case 3.3 (lines 13–14), the number of received packets is higher than the estimated number of expected packets (i.e., $\pi_{b,r}^{s}>1/t_{\mathrm{int}}^{s}$) and the transmission interval is reduced by adding 1 to the inverse (line 14). Case 3.4 (lines 15–16) represents the inverse case, where fewer packets are received than estimated, and the transmission interval is increased by subtracting 1 from the inverse (line 16). Finally, the next transmission time is calculated as the last successful transmission plus the newly estimated transmission interval (line 17). In essence, the algorithm is iterative, and as more information about successful and failed transmissions becomes available, the estimate of $t_{\mathrm{int}}^{s}$ will become more accurate.

### 4.4. RAW Slot Assignment 

The second main step of TAROA determines the set of RAW slots $R^{b}$ to initialize in the next beacon interval *b*, as well as for each of the RAW slots their duration $t^{r}$, and the assigned stations $S^{r}$. These RAW parameters are selected based on the previously determined estimation of the transmission interval $t_{\mathrm{int}}^{s}$ and next transmission time $t_{\mathrm{next}}^{s}$. As shown in Algorithm 2, this is a two-step process: (i) select a subset of stations with pending packets to be assigned to a RAW slot during the upcoming beacon interval (lines 2–8); and (ii) partition the selected stations among the available number of RAW slots and determine the slot duration (lines 9–14).
**Algorithm 2:** RAW parameter configuration for beacon interval *b*
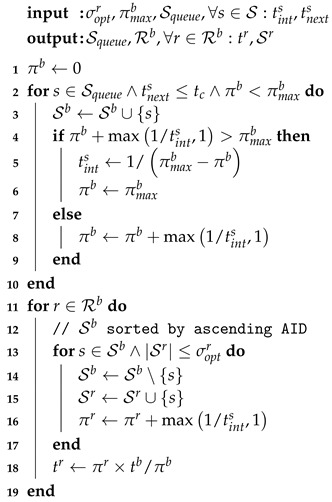


In the first part (lines 2–8), the algorithm iterates over all stations *s*, according to ascending last successful transmission time $t_{\mathrm{succ}}^{s}\left[0\right]$ (i.e., $S_{\mathrm{queue}}$) until the next station has a next estimated transmission time greater than the current time (i.e., $t_{\mathrm{next}}^{s}>t_{c}$) or the maximum allowed number of packet transmissions has been reached (i.e., $\pi^{b}\geq \pi_{\max}^{b}$) (line 2). The station is first added to the set of stations allowed to transmit during the beacon interval $S^{b}$ (line 3). Then, if the station is estimated to transmit more than one packet per beacon interval (i.e., $t_{\mathrm{int}}^{s}<1$) and its estimated number of transmissions will exceed the maximum packet transmissions in *b* (line 4), then it is allowed to transmit part of its packets and its estimated transmission interval is updated accordingly (line 5). Otherwise, the station is expected to be able to transmit all of its packets and $\pi^{b}$ is increased with the number of packets *s* is estimated to transmit in the beacon interval (line 8).

At this point, it should be noted that all slots within a RAW group have the same duration and only stations with sequential AID can be assigned to the same group. Moreover, the optimal duration of a slot depends on the number of stations assigned to it, as well as their data rates, number of queued packets, and packet payload sizes. As such, for simplicity, we assume throughout the remainder of this paper that each RAW group has exactly one slot, allowing all slots to have a different size. Overcoming this problem is possible by selecting a worst-case slot duration (suboptimal) or performing dynamic AID reassignment. The latter is expected to increase optimality, but falls outside the scope of this paper.

In the second part (lines 9–14), stations are assigned to RAW slots $r\in R^{b}$ according to increasing AID (as only sequential AID can be assigned to the same group) until the number of stations assigned to the slot are greater or equal to the optimal number of assigned stations (i.e., $\left|S^{r}\right|>\sigma_{\mathrm{opt}}^{r}$) (line 10). How to calculate this optimum is explained in more detail in Section 5. The number of packets to be transmitted by *s* is added to the expected number of packet transmissions in *r* (line 13). Finally, the optimal duration $t^{r}$ of the slot is determined based on the number of expected packet transmission $\pi^{r}$ (line 14).

## 5. Optimal Input Parameter Derivation

In addition to information about past transmissions, the TAROA algorithm takes two additional input parameters: (i) the optimal number of stations in one RAW slot $\sigma_{\mathrm{opt}}^{r}$; and (ii) the maximum number of packet transmissions in one beacon interval $\pi_{\max}^{b}$. The optimal value for each of these parameters can be derived analytically or experimentally for a given network topology and configuration. This section describes how to derive these values.

### 5.1. Optimal Number of Stations in One RAW Slot 

As the idea of RAW is to limit the number of station contending for the channel, an appropriate number of stations $\sigma_{\mathrm{opt}}^{r}$ that share a RAW slot should be determined in order to strike the proper balance between contention and channel utilization.

Several researchers have proposed analytic models to calculate throughput of 802.11ah networks with a variable number of stations [23,26,27,28,29,30]. These models can be used to obtain a value for $\sigma_{\mathrm{opt}}^{r}$. However, they all assume an ideal channel, which does not take the capture effect into account. In real life, the capture effect allows the receiver to still decode certain packets in case of a collision, when the received power of the colliding packets differs significantly. This significantly increases throughput, resulting in pessimistic throughput estimations of existing analytical models. This is especially true for low data rates, where packets are more easily captured as lower signal-to-interference power ratio is required. Currently, our 802.11ah ns-3 simulator implementation inherits the partially implemented capture effect feature from the standard 802.11 module in ns-3 version 3.23, which allows a packet to be captured when it arrives at the receiver before a colliding packet with lower receive power, while both packets will be dropped if the colliding packet has higher power. Therefore, in order to get more realistic results, we derive $\sigma_{\mathrm{opt}}^{r}$ through simulation rather than analytical models. We calculate $\sigma_{\mathrm{opt}}^{r}$ as the number of stations that achieve the highest throughput in saturated state.

As 802.11ah focuses on IoT and M2M scenarios, 1 and 2 MHz channel bandwidths are most commonly used, with the data rate ranging from 0.15 to 7.8 Mbps. Table 3 lists the optimal $\sigma_{\mathrm{opt}}^{r}$ for different data rates (0.15, 0.6, 2.6 and 7.8 Mbps) and packet sizes (16, 64, 256 and 1024 bytes), as derived using simulation results. The simulations were performed using the same PHY and MAC parameters as used in Section 6 (cf. Table 4). All the stations are in saturated state and there are no hidden nodes among them. The RAW slot duration used in the simulation equals the beacon interval (100 ms). As Zheng et al. [30] reveal that the amount of wasted channel time in a RAW slot supporting cross-slot boundary is bounded by the duration of the AIFS (316 μs), the value of $\sigma_{\mathrm{opt}}^{r}$ obtained from these simulation results is suitable for RAW slots with different durations as well. The simulation results show that the optimal value of $\sigma_{\mathrm{opt}}^{r}$ is quite small for high data rates and large packet payload sizes, even becoming 1 station per slot for packets of 1024 bytes. As the data rate and packet payload size decrease, $\sigma_{\mathrm{opt}}^{r}$ becomes larger, reaching 180 stations for data rate 0.15 Mbps and packet payload size 16 bytes. The larger $\sigma_{\mathrm{opt}}^{r}$ for lower data rates is mainly caused by the capture effect, as low data rates need lower SIR (signal-to-interference power ratio) in order to capture the collided packets. Since only stations with sequential AID can be assigned to the same group (and therefore slot), we use $\sigma_{\mathrm{opt}}^{r}$ as the maximum number of stations that can be allocated in one RAW slot. As AID assignment may be suboptimal, this may bring about slight performance degradation as the actual number of stations that can be assigned to one RAW slot may be smaller than $\sigma_{\mathrm{opt}}^{r}$. This could be alleviated through AID reassignment.

As mentioned above, we derive the optimal number of stations $\sigma_{\mathrm{opt}}^{r}$ in one RAW slot based on the saturated state. In an unsaturated state, which is the most common case in reality, contention decreases. In this unsaturated case, allowing $l>\sigma_{\mathrm{opt}}^{r}$ stations to contend in each RAW slot will achieve the same throughput, and at the same time achieve lower latency. This has been demonstrated through simulation in our own previous work [1] and by the analytical model presented by Duffy et al. [33]. In this article, we use $\sigma_{\mathrm{opt}}^{r}$ to maximize throughput in both saturated and unsaturated scenarios. Obtaining a larger $\sigma_{\mathrm{opt}}^{r}$ based on traffic load and AID re-assignment to further optimize latency as well as throughput is considered future work.

Even though the derived optimal value of $\sigma_{\mathrm{opt}}^{r}$ depends on the packet size, TAROA does support packet size variability, due to the use of cross boundary slots. An average packet size can be assumed, and some degree of variability in packet size (and thus transmission rate) is averaged out over multiple slots.

### 5.2. Maximum Number of Packet Transmissions in One Beacon Interval

Let $S_{\max}$ denote the throughput that can be achieved by assigning $\sigma_{\mathrm{opt}}^{r}$ stations in one RAW slot as shown in Algorithm 2, the maximum number of packet transmissions in one beacon interval $\pi_{\max}^{b}$ is then calculated as follows:

(4) $$\pi_{\max}^{b}=\frac{S_{\max}\times t^{b}}{L},$$

where *L* is the average packet payload size. When the channel is fully utilized (i.e., $\pi_{\max}^{b}$ packets scheduled to be transmitted in one beacon interval), the duration $t_{\mathrm{avg}}^{r}$ of a RAW slot *r* equaling the time needed on average to finish all packet transmissions assigned to *r*, can be calculated as:

(5) $$t_{\mathrm{avg}}^{r}=\frac{\pi^{r}\times t^{b}}{\pi_{\max}^{b}}.$$

**Figure 5:**
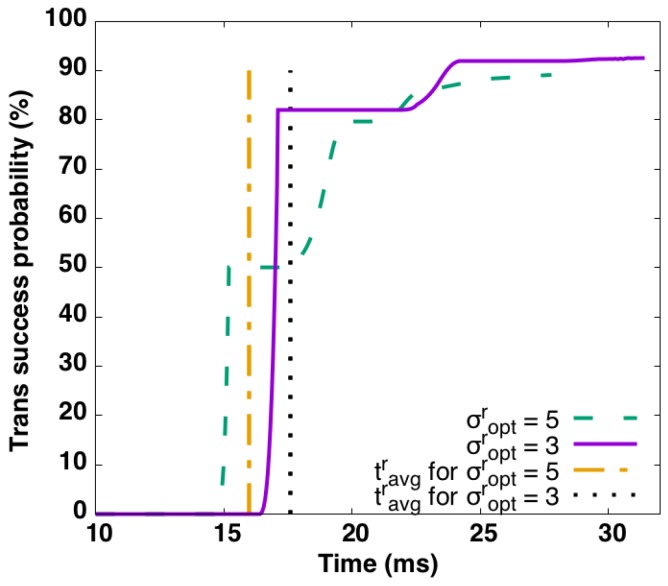
Transmission success probability as a function of time for $\sigma_{opt}^{r}=5$, 3, which are used for data rate 0.6 Mbps with packet size 64 and 256 bytes, respectively.

However, as 802.11ah employs EDCA/DCF inside RAW slots, these calculations are based on average back-off times, and only guarantee successful transmission up to a certain probability. The transmission success probability can be evaluated by the model proposed by Khorov et al. [23], which assumes each station only has one packet to transmit during their assigned RAW slot. Given the sensor use case considered in this article, the assumption that each station will at most transmit one packet per beacon interval (generally set to 100 ms) can generally be assumed to hold. Based on this model, Figure 5 depicts how the transmission success probability changes over time using a data rate of 0.6 Mbps with packet sizes 64 and 256 bytes, respectively. For a packet size of 64 bytes, $\sigma_{\mathrm{opt}}^{r}$ is 5 and $t_{\mathrm{avg}}^{r}$ is 16.53 ms, while $\sigma_{\mathrm{opt}}^{r}$ is 3 and $t_{\mathrm{avg}}^{r}$ is 17.59 ms for a packet size of 256 bytes. The figure clearly shows that the transmission success probability is only 50% and 82% respectively when $t_{\mathrm{avg}}^{r}$ is used as RAW slot duration.

The solution is to increase $t_{\mathrm{avg}}^{r}$ to get a higher transmission success probability. However, $\pi_{\max}^{b}$ will also become lower and more channel time will be wasted, which, in turn, degrades performance. However, the model of Khorov et al. [23] considers cross slot boundary transmission as forbidden. As the cross slot boundary feature of 802.11ah does allow transmissions to continue after the current RAW slot ends, and our algorithm only estimates traffic intensity based on transmission success at the end of the beacon interval (and not at the end of the slot), the actual transmission success probability when using the optimally calculated $\pi_{\max}^{b}$ is much higher in reality than depicted in Figure 5. As such, we propose the use of the cross slot boundary feature in combination with the slot duration calculated in Equation (5) when the channel is fully utilized. Moreover, we assume all slots are RAW capable, and therefore the entire channel time is occupied by RAW. When the channel is not fully utilized, $\pi^{b}<\pi_{\max}^{b}$ packet transmissions are allowed in one beacon. The RAW slot *r* then has the following duration:

(6) $$t^{r}=\frac{\pi^{r}\times t^{b}}{\pi^{b}}>t_{\mathrm{avg}}^{r}.$$

As such, the transmission success probability is improved. Therefore, instead of looking for an optimal $\pi_{\max}^{b}$ and $t^{r}$ that can balance transmission success probability and wasted channel time, we simply obtain them with Equations (4) and (5) by taking advantage of the cross-slot boundary feature and allowing the entire channel time to be occupied by RAW slots.

## 6. Performance Evaluation and Discussion

### 6.1. Simulation Setup

All evaluations are performed using our previously developed 802.11ah ns-3 module [2], based on ns-3 version 3.23. We consider two IoT scenarios, where sensors periodically monitor the environment and send the resulting data to a server (via the AP). Different sensors may have different monitoring and transmission intervals, which may change over time. The transmission interval of sensors in an IoT network follows a uniform distribution. The ratio between any two sensors’ transmission interval in an experiment is never higher than 20. The default PHY and MAC layer parameters used in our simulation are shown in Table 4. Given the low-power nature of battery powered sensors, the PHY layer parameters are configured based on the low-power 802.11ah radio hardware prototype developed by Ba et al. [15], with a transmission power of 0 dBm, a gain of 0 dBi (for both sensor and AP), and noise figure of 6.8 dB. As Section 5.1 indicates, the data rate and payload size impose an impact on the performance of TAROA. Therefore, two scenarios are considered: (i) high-throughput (HT) using MCS8 with 2 MHz bandwidth (data rate 7.8 Mbps) and payload size 256 bytes; and (ii) low-throughput (LT) using MCS1 with 1 MHz bandwidth (data rate 0.15 Mbps) and payload size 64 bytes. The two scenarios are hence referred to as HT and LT, respectively. For each scenario, TAROA is evaluated both with and without hidden nodes, with the stations randomly placed around the AP in a circle of 50 and 100 m, respectively, for the HT scenario, and 200 and 450 m, respectively, for the LT scenario. Taking into account the IoT scenario we evaluate, in which the traffic of each station is quite low, a small buffer size can be used. Therefore, the size of the stations’ transmit queues is configured to be 10 packets. Thus, packets can be dropped during simulation due to buffer overflow. No rate control algorithm (RCA) is used at the MAC layer.

RAW performance is evaluated in terms of two metrics: throughput, latency and packet loss. Throughput is calculated as the average number of successfully received payload bytes by the AP per second. Latency is defined as the average time between a packet entering the transmit queue of the station and being received by the AP. Packet loss represents the ratio between the number of packets not received by AP, and the number of packets sent by all stations. Each simulation runs 600 s. This simulation time is long enough as each station transmits packets with a certain (predictable) frequency, the steady-state simulation result is achieved after less than 100 s in all experiments. All results are averaged over 10 iterations, with the variability of results over these iterations quantified using the standard deviation (SD).

### 6.2. Static Traffic Patterns

This section evaluates the performance of TAROA for different traffic loads and numbers of stations in a static network, which means that stations stay active from the beginning to the end of the simulation and do not change their transmission interval. Three different total traffic loads are simulated for each scenario, i.e., *T* = {0.75, 0.85, 1.2} Mbps for the HT scenario, and *T* = {0.095, 0.11, 0.15} Mbps for the LT scenario. Each station *s* randomly and uniformly chooses an integer value $v^{s}$ in the interval [1,20]; therefore, the total value for station set *S* is

$$V^{S}=\sum_{s\in S}v^{s}.$$

For each station *s* its traffic load is calculated as:

$${\hat{T}}^{s}=\frac{T\times v^{s}}{V^{S}}.$$

Its actual packet transmission interval is subsequently calculated as:

$${\hat{t}}_{\mathrm{int}}^{s}=\frac{\mathrm{PayloadSize}\times 8}{{\hat{T}}^{s}\times {\hat{t}}^{b}},$$

where ${\hat{t}}^{b}$ represents the beacon interval time. Given the packet payload size and data rate, the maximum throughput that can be achieved is about 1.049 and 0.124 Mbps for HT and LT, respectively. As such, *T* = {1.2, 0.15} Mbps represents a saturated state (η = 114%, 120%), *T* = {0.85, 0.11} Mbps represents a medium traffic load (η = 81%, 88%), and *T* = {0.75, 0.095} Mbps results in low traffic load (η = 71%, 76%). Here, η denotes the ratio between traffic load and maximum practical throughput that can be achieved. Together with the number of stations, η is used to describe the density of the network. A network without and with hidden nodes is both simulated. As a benchmark, we compare to the traditional 802.11 channel access method based on EDCA/DCF and fixed RAW groups.

#### 6.2.1. Without Hidden Node

**Figure 6:**
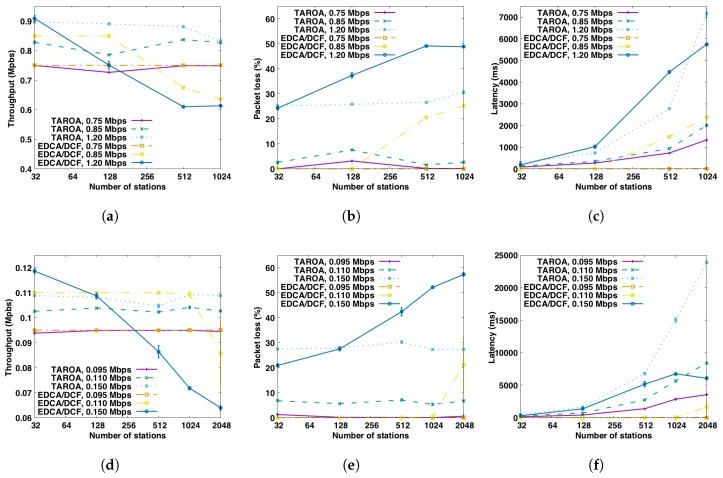
Performance comparison between TAROA and EDCA/DCF for both the HT and LT scenarios without hidden nodes for different traffic loads and number of stations. (**a**) throughput, HT; (**b**) packet loss, HT; (**c**) latency, HT; (**d**) throughput, LT; (**e**) packet loss, LT; (**f**) latency, LT.

Figure 6 depicts the performance of the evaluation metrics (throughput, latency and packet loss) when there are no hidden nodes in the networks for both the HT and LT scenario. For the HT scenario, Figure 6a clearly shows that TAROA scales much better than EDCA/DCF in dense networks in terms of throughout. For traffic load *T* = 1.2 Mbps, EDCA/DCF achieves throughput 0.909±0.010 Mps and TAROA gets 0.898±0.003 Mbps for 32 stations. However, as the number of stations increases to 1024, throughput of EDCA/DCF dramatically decreases to 0.613±0.001 Mbps (i.e., −32%), and TAROA still achieves 0.832±0.010 Mbps (i.e., only −7%). With traffic load *T* = 0.85 Mbps, throughput of EDCA/DCF drops by 25% between 128 and 1024 stations, while there is no significant drop for TAROA. For the lowest traffic load *T* = 0.75 Mbps, there is no significant difference between TAROA and EDCA/DCF.

Figure 6b suggests that the packet loss of TAROA is much less than EDCA/DCF in dense networks, which is quite straightforward since TAROA achieves much higher throughput than EDCA/DCF. For traffic load *T* = 1.2 Mbps, packet loss of EDCA/DCF increases from 24.24%±0.87% to 48.87%±0.12% when the number of stations increases from 32 to 1024, while packet loss caused by collisions increases from 0.16%±0.01% to 19.81%±0.24%. While, for TAROA, packet loss increases from 25.15%±0.28% to 30.62%±0.71%. With traffic load *T* = 0.85 Mbps, packet loss of EDCA/DCF is 20.51%±0.62% and 25.26%±0.10%, respectively, for 512 and 1024 stations, 7.43%±0.27% and 14.62%±0.13% packets are lost due to collisions. While only 1.58%±0.09% and 2.62%±0.27% packet loss occurs with TAROA. For the lowest traffic load *T* = 0.75 Mbps, there is no significant difference between TAROA and EDCA/DCF. When using TAROA, no packet loss due to collisions occurred.

In terms of latency, Figure 6c shows that TAROA also outperforms EDCA/DCF in most dense networks. The latency can be affected by different aspects, and these factors jointly determine the overall latency. First, fierce channel contention increases the time needed to successfully delivery packets to the AP. Second, in TAROA, RAW schedules stations to contend for the channel only at certain times, which extends the queuing time of packets and in turn increases latency. Especially when the traffic load is low and there is little contention, this results in more packets accumulating in the transmit queue. For medium and high traffic load (i.e., $T=\left\{0.85,1.2\right\}$ Mbps), TAROA usually results in improved latency compared to EDCA/DCF for 256 stations or more, due to high contention in the latter. EDCA/DCF only outperforms TAROA for T=1.2 Mbps and 1024 stations, as EDCA/DCF results in a huge amount of dropped packets, artificially lowering the latency. For low traffic loads (i.e., T=0.75 Mbp), however, EDCA/DCF provides a better latency, due to the slotted nature of RAW.

Performance for the LT scenario is depicted in Figure 6d–f, revealing similar conclusions in terms of throughput and packet loss scalability as for HT. For a high load of T=0.150 Mbps, EDCA/DCF throughput drops around to 46% between 32 and 2048 stations, while that of TAROA remains constant around 0.11±0.0002 Mbps. The figure, however, also shows that EDCA/DCF scales better for low data rates, as throughput under medium load in the LT scenario only decreases with more than 1024 stations (in contrast to 128 in HT). In this case, throughput drops 23% between 1024 and 2048 stations. In terms of packet loss, for traffic load T=0.150 Mbps and T=0.110 Mbps, TAROA performs better than EDCA/DCF withe more than 128 and 1024 stations, respectively. However, as EDCA/DCF scales better for low data rates and TAROA needs to estimate the traffic load of each station, using TAROA results in more packet loss for traffic load T=0.110 Mbps with less than 1024 stations. In addition, for low traffic load T=0.095 Mbps, up to 1.25%±0.13% packets are lost for TAROA, and there is no packet loss for EDCA/DCF. Packet loss caused by collisions for EDCA/DCF increases as the number of stations increases for traffic load T=0.150 Mbps and T=0.110 Mbps. In a network with 2048 stations, it goes up to 40.07%±0.54% and 18.62%±9.85%, respectively, while no packets are lost due to collision when using TAROA.

For the LT scenario, EDCA/DCF always results in better latency, due to the slotted nature of RAW and the large quantities of packet loss of EDCA/DCF in very dense networks.

**Figure 7:**
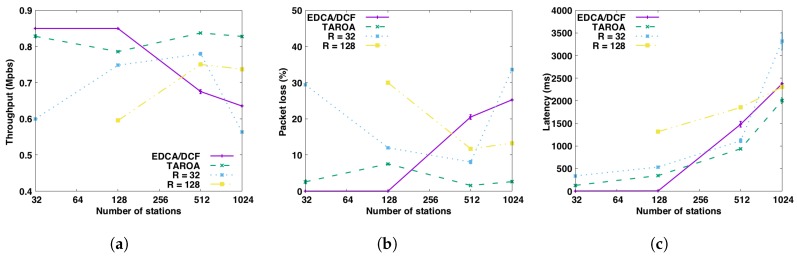
Performance comparison between TAROA, EDCA/DCF and fixed number of RAW groups (R) for the HT scenario (*T* = 0.85 Mbps) without hidden nodes. (**a**) throughput; (**b**) packet loss; (**c**) latency.

Figure 7 zooms in on the performance (throughput, latency and packet loss) in the HT scenario with a fixed number of RAW groups (R=32, 128) under traffic load of 0.85 Mbps, each RAW group has the same number of stations. The results suggest that the performance of a fixed number of RAW groups varies as the number of stations changes. This conclusion is further supported by Table 5. As such, there is no one optimal fixed RAW group configuration for all different network topologies. This proves the further need for a dynamic RAW configuration algorithm, such as TAROA, which achieves good performance regardless of the network topology.

#### 6.2.2. With Hidden Nodes

**Figure 8:**
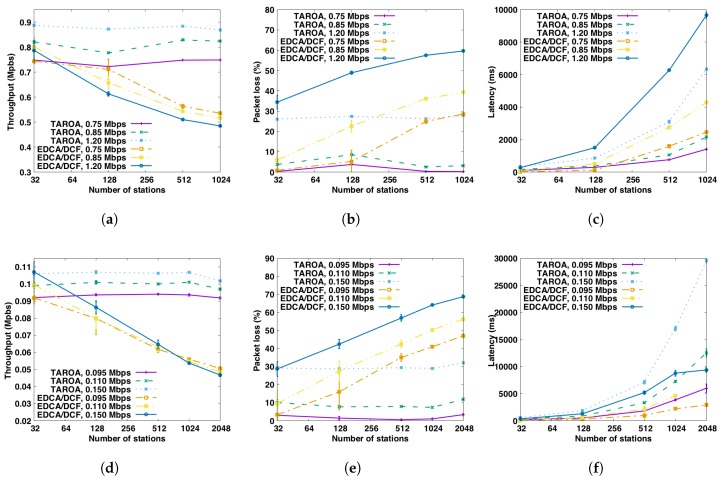
Performance comparison between TAROA and EDCA/DCF for both the HT and LT scenario with hidden nodes for different traffic loads and number of stations. (**a**) throughput, HT; (**b**) packet loss, HT; (**c**) latency, HT; (**d**) throughput, LT; (**e**) packet loss, LT; (**f**) latency, LT.

In this section, we study the impact of hidden nodes imposed on performance by extending the maximum distance between the AP and the stations to 100 m for the HT and 450 m for the LT scenario. The results are shown in Figure 8. This clearly reveals that when hidden nodes are present, TAROA gains additional advantage over EDCA/DCF, showing its potential to successfully avoid hidden nodes. While TAROA shows stable throughput performance for all traffic loads as a function of the number of stations, EDCA/DCF suffers more heavily. Even for low traffic loads, the throughput when using EDCA/DCF degenerates fast with more than 32 nodes in both the HT scenario and the LT scenario. For HT, EDCA/DCF shows a throughput drop between 32 and 1024 stations of up to 38%. For LT, its throughput even drops over 56%. Correspondingly, hidden nodes significantly increase the packet loss of EDCA/DCF, TAROA get less packet loss for all three of the traffic loads with more than 32 stations. With hidden nodes, collisions result in a very small amount of packet loss for TAROA, up to 0.003%±0.002%. In the HT scenario, TAROA consistently outperforms EDCA/DCF in terms of latency. For LT, this is not always the case, due to the aforementioned reasons. For traffic load 1.2 Mbps with 1024 stations, in which the traffic is overloaded and stations get less transmission opportunity than they require, the results show that better performance is achieved with hidden nodes than without hidden nodes. This is due to overestimation of the transmission interval.

#### 6.2.3. Transmission Interval Estimation Accuracy

**Figure 9:**
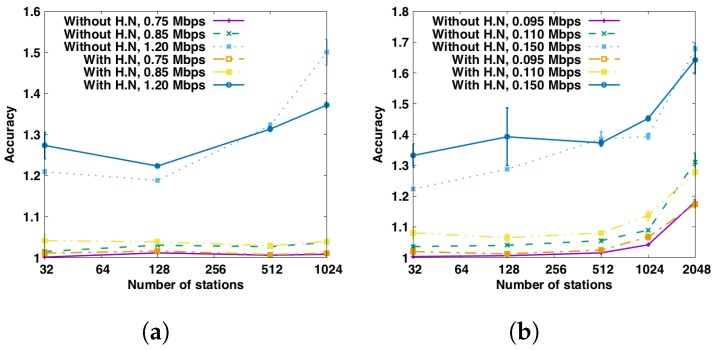
Accuracy of transmission interval estimation with and without hidden nodes (H.N) for both HT and LT scenarios. (**a**) HT scenario; (**b**) LT scenario.

Figure 9 depicts the accuracy of transmission interval estimation for both HT and LT scenarios with and without hidden nodes (H.N.). The “accuracy” represents the ratio between the estimated transmission interval and the real transmission interval, averaged over all stations. As such, a value equal to 1 means no estimation error, higher than 1 means overestimation and lower than 1 means underestimation. For the HT scenario, the estimation is quite accurate for traffic load *T* = {0.85, 0.75} Mbps (η=81%,71%) as the accuracy is close to 1. While for high traffic load *T* = 0.12 Mbps (η=114%), the transmission interval is highly overestimated, since the traffic is overloaded and each station get less transmission opportunity than it requires. The same conclusion can be drawn for the LT scenario.

### 6.3. Dynamic Number of Stations

In this section, we study the ability of TAROA to adapt to changes in the topology (i.e., number of associated stations) over time. The total traffic load, 1.275 Mbps for HT and 0.1425 Mbps for LT, is distributed among 1536 stations in the same way as mentioned in Section 6.2. The simulation only starts with 1024 associated stations and a traffic load of 0.85 Mbps for the HT scenario, and 1024 associated stations and a traffic load of 0.095 Mbps for LT scenario. A Poisson distribution then is used to model the arrival (i.e., association) and departure of stations, where a higher Poisson rate λ results in faster changes.

Table 6 lists the average throughput of TAROA and EDCA/DCF when stations join and leave the network with Poisson rates 0.1, 1 and 5 every second. The results show that, as expected, EDCA/DCF adapts quite well to network dynamics. TAROA suffers more significantly, as it constantly needs to adapt the RAW configuration as the network topology changes. Moreover, estimating the transmission interval of a newly joined station takes a few packet transfers to converge. Nevertheless, TAROA shows resilience to topology change and only suffers significantly when the Poisson rate is 5, which corresponds to on average five stations joining and leaving the network every second. We argue that five stations joining and leaving the network every second is an unrealistically high number for the IoT scenario under investigation. As such, TAROA adapt well under realistic dynamic conditions. Overall, TAROA outperforms EDCA/DCF in all scenarios, except LT without hidden nodes. However, in this scenario (LT with 1024 stations and 0.095 Mbps), EDCA/DCF also outperformed TAROA in the static case. To summarize, even if EDCA/DCF is inherently more resilient to topology changes than a slotted mechanism like RAW, TAROA is still able to outperform EDCA/DCF under traffic conditions that correspond to static scenarios where TAROA is better. It should be pointed out that EDCA/DCF simulations with hidden nodes at Poisson rate 1 and 5, respectively, for LT scenarios use an association time-out of 10 s. This was done as the default association time-out of 0.5 s prevented stations using EDCA/DCF from properly associating with the AP in these two scenarios, which resulted in continuous performance degradation.

### 6.4. Dynamic Traffic

In addition to stations changing over time, we also evaluate the performance when the number of active stations does not change, but instead their transmission interval changes over time. For the HT scenario, the network consists of 1024 stations with a total traffic load of 0.85 Mbps at the start of simulation, while for LT there are 1024 stations with a total traffic load of 0.095 Mbps at the start of simulation. Traffic distribution among stations is done according to the method detailed in Section 6.2. Every second, a random set of stations is selected that will change their transmission interval, according to a Poisson distribution with rate λ=5. For each selected station, the change in transmission interval Δ is chosen uniformly at random as a percentage between $\left[-10,10\right]$, $\left[-20,20\right]$, or $\left[-50,50\right]$.

Table 7 lists the average throughput for both EDCA/DCF as well as TAROA. The conclusions drawn in the previous section also apply here. Specifically, TAROA outperforms EDCA/DCF in all dynamic traffic scenarios if it also outperforms EDCA/DCF under similar static conditions (i.e., in all scenarios expect LT with 1024 stations and a starting traffic load of 0.095 Mbps). Moreover, changes in traffic load result in far less degradation of throughput than changes in topology, both for EDCA/DCF and TAROA.

## 7. Conclusions

In this paper, we propose a novel traffic-adaptive RAW optimization algorithm (TAROA) to adjust the RAW parameters in real time based on the current traffic conditions, optimized for sensor networks with mainly upstream traffic in which each station is assumed to transmit packets with a certain (predictable) frequency. The TAROA algorithm improves upon the state of art in three ways, including supporting dynamic and heterogeneous traffic conditions, only using information available on the AP side and real-time execution. The combination of these three factors allows TAROA to be deployed in realistic environment. The TAROA algorithm is executed at each target beacon transmission time (TBTT), it first estimates the packet transmission interval of each station only based on packet transmission information obtained during the last beacon interval, and then determines the RAW parameters and assigns stations to RAW slots based on this estimated transmission frequency.

The simulation results reveal three key points on TAROA. First, it scales much better than EDCA/DCF in dense networks in terms of throughput under static traffic conditions. Second, it is resilient to dynamic traffic conditions in which topology or transmission interval change over time. In the scenario under evaluation, TAROA only starts to experience throughput degradation when topology changes at Poisson rate 5. Third, TAROA gains additional advantages over EDCA/DCF when hidden nodes are present under both static and dynamic traffic conditions. Hidden nodes highly degrade the performance of EDCA/DCF, while TAROA is hardly affected. In summary, for dense IoT networks, TAROA can easily adapt to current traffic conditions in real time and significantly improves throughput.

In future work, we will further improve the performance of the TAROA algorithm, and, in particular, the traffic estimation under high load. Moreover, TAROA will be extended to support adaptive MCS.

## Figures and Tables

**Table 1:** Classification of existing IEEE 802.11ah station grouping algorithms and models in terms of supported upstream traffic conditions, optimization objective, and used algorithmic method.

Reference	Traffic Conditions	Objective	Algorithmic Method
Yoon et al. [18]	1 packet, homogeneous	contention	set partitioning (hidden nodes)
Dong et al. [19]	static, heterogeneous	contention	set partitioning (node location)
Damayanti et al. [20]	1 packet, homogeneous	contention	set partitioning (hidden nodes)
Chang et al. [21]	static, heterogeneous	throughput	set partitioning (traffic demand)
Wang et al. [22]	1 packet, homogeneous	energy	probabilistic analytical model
Khorov et al. [23]	1 packet, homogeneous	throughput	Markov chains
Qutab-ud et al. [24]	saturated, homogeneous	throughput	set partitioning (back-off timer)
Bel et al. [25]	1 packet, homogeneous	energy	multi-objective game theory
Park et al. [26]	saturated, homogeneous	throughput	maximum likelihood estimation
Raeesi et al. [27,28]	saturated, homogeneous	throughput & energy	probabilistic analytical model
Zheng et al. [29,30]	saturated, homogeneous	throughput	Markov chains
Ogawa et al. [31]	saturated, homogeneous	throughput & energy	set partitioning (uniform random)

**Table 2:** Variables and notations introduced in the algorithm description.

**General Variables**	**Description**
S	Set of all stations
$S_{\mathrm{queue}}$	Queue of stations sorted by increasing next transmission time $t_{\mathrm{next}}^{s}$
$t_{c}$	Current time ${}^{⋆}$
**Variables of Beacon Interval *b***	**Description**
$R^{b}$	Set of RAW slots in beacon interval *b*
$t^{b}$	Total beacon interval time minus beacon transmission time
$S^{b}$	Set of stations that are allowed to transmit in the beacon interval b
$\pi_{\max}^{b}$	Theoretical maximum number of packet transmissions in beacon interval *b*
$\pi^{b}$	Number of packet transmissions in beacon interval *b*
**Variables of RAW Slot $r\in R^{b}$**	**Description**
$S^{r}$	Set of stations assigned to RAW slot *r*
$\sigma_{\mathrm{opt}}^{r}$	Optimal number of stations in RAW slot *r* based on throughput
$\pi^{r}$	Number of packet transmissions in RAW slot *r*
$t^{r}$	RAW slot duration
**Variables of Station s∈S**	**Description**
$\pi_{b,r}^{s}$	Number of packets received by the AP in RAW slot *r* of beacon interval *b* from station *s*
$\pi_{\mathrm{failed}}^{s}$	Number of consecutive failed transmissions of station $S^{i}$
$t_{\mathrm{next}}^{s}$	Estimated next transmission time ${}^{⋆}$
$t_{\mathrm{int}}^{s}$	Estimated transmission interval ${}^{⋆}$
${\hat{t}}_{\mathrm{int}}^{s}$	Real transmission interval ${}^{⋆}$
$t_{\mathrm{succ}}^{s}\left[0\right]$	Last successful transmission time ${}^{⋆}$
$t_{\mathrm{succ}}^{s}\left[1\right]$	Previous to last successful transmission time ${}^{⋆}$
$\pi_{\mathrm{trans}}^{s}\left[0\right]$	Last transmission result, success or failure
$\pi_{\mathrm{trans}}^{s}\left[1\right]$	Previous to last transmission result, success or failure

${}^{⋆}$ Expressed as a multiple of number of beacon intervals.

**Table 3:** Optimal number of stations in one RAW slot $\sigma_{\mathrm{opt}}^{r}$ for different data rates and packet payload sizes.

	Packet Payload Size (Bytes)			
Data Rate (Mbps)	16	64	256	1024
**0.15**	180	128	32	6
**0.60**	5	5	3	1
**2.6**	5	5	5	1
**7.8**	2	2	2	1

**Table 4:** Default parameter values used in the simulation experiments.

**(a) Default PHY Parameters**	
**Parameters**	**Values**
Frequency (MHz)	868
TX power (dBm)	0
TX gain (dB)	0
RX gain (dB)	0
Noise Figure (dB)	6.8
Coding method	BCC
Propagation model	Outdoor, macro [9]
Error rate model	YansErrorRate
**(b) Default MAC Parameters**	
**Common Parameters**	**Values**
Duration of AIFS (μs)	316
RTS/CTS	not enabled
Beacon interval (ms)	100
Cross slot boundary	enabled
Station distribution	random
Rate control algorithm	constant
Size of transmit queue (packets)	10
Max/min traffic ratio between stations	20
**High-Throughput (HT) Parameters**	**Values**
Wi-Fi mode	MCS8, 2 MHz
Payload size (bytes)	256
Non-hidden node topology radius (m)	50
Hidden node topology radius (m)	100
**Low-Throughput (LT) Parameters**	**Values**
Wi-Fi mode	MCS1, 1 MHz
Payload size (bytes)	64
Non-hidden node topology radius (m)	200
Hidden node topology radius (m)	450

**Table 5:** Comparison of throughput of different fixed RAW groups (R), EDCA/DCF and TAROA under various traffic loads (T) and number of stations (N).

**T (Mbps)**	**HT Scenario, Without Hidden Nodes**											
	***n* = 128**				***n* = 512**				***n* = 1024**			
	**R = 32**	**R = 128**	**EDCA/DCF**	**TAROA**	**R = 32**	**R = 128**	**EDCA/DCF**	**TAROA**	**R = 32**	**R = 128**	**EDCA/DCF**	**TAROA**
0.75	0.70	0.52	0.75	0.73	0.34	0.34	0.75	0.75	0.28	0.35	0.75	0.75
0.85	0.75	0.60	0.85	0.79	0.78	0.75	0.68	0.84	0.56	0.74	0.64	0.83
1.20	0.86	0.72	0.75	0.89	0.59	0.58	0.61	0.88	0.44	0.55	0.61	0.83
**T (Mbps)**	**LT Scenario, Without Hidden Nodes**											
	***n* = 512**				***n* = 1024**				***n* = 2048**			
	**R = 32**	**R = 128**	**EDCA/DCF**	**TAROA**	**R = 32**	**R = 128**	**EDCA/DCF**	**TAROA**	**R = 32**	**R = 128**	**EDCA/DCF**	**TAROA**
0.095	0.074	0.087	0.095	0.095	0.093	0.092	0.095	0.095	0.069	0.087	0.095	0.094
0.110	0.103	0.095	0.110	0.102	0.104	0.102	0.109	0.104	0.075	0.097	0.086	0.103
0.150	0.115	0.109	0.086	0.105	0.107	0.115	0.072	0.109	0.063	0.108	0.064	0.109

**Table 6:** Comparison of throughput of EDCA/DCF and TAROA under three different intensities of dynamic number of stations.

λ	**HT Scenario**			
	**Without Hidden Nodes**		**With Hidden Nodes**	
	**EDCA/DCF**	**TAROA**	**EDCA/DCF**	**TAROA**
0.1	0.63010±0.00142	0.83621±0.00119	0.51374±0.00237	0.83027±0.00134
1	0.62942±0.00165	0.81656±0.00193	0.51080±0.00379	0.76800±0.00274
5	0.60546±0.00209	0.74428±0.00305	0.48361±0.00313	0.67500±0.00328
λ	**LT Scenario**			
	**Without Hidden Nodes**		**With Hidden Nodes**	
	**EDCA/DCF**	**TAROA**	**EDCA/DCF**	**TAROA**
0.1	0.09706±0.00003	0.09568±0.00036	0.05440±0.00140	0.09476±0.00058
1	0.09485±0.00004	0.09106±0.00045	0.04964±0.00191 ${}^{⋆}$	0.08864±0.00053
5	0.09712±0.00004	0.07749±0.00029	0.03284±0.00146 ${}^{⋆}$	0.07431±0.00058

${}^{⋆}$ indicates that simulation uses association time out 10 s instead of 0.5 s.

**Table 7:** Comparison of throughput of EDCA/DCF and TAROA under three different intensities of dynamic station transmission intervals.

Δ(%)	**HT Scenario**			
	**Without Hidden Nodes**		**With Hidden Nodes**	
	**EDCA/DCF**	**TAROA**	**EDCA/DCF**	**TAROA**
$\left[-10,10\right]$	0.63673±0.00216	0.82591±0.00135	0.51863±0.00501	0.82441±0.00199
$\left[-20,20\right]$	0.63741±0.00265	0.83399±0.00139	0.52047±0.00344	0.82843±0.00065
$\left[-50,50\right]$	0.64063±0.00403	0.83163±0.00085	0.52180±0.00398	0.82681±0.00154
Δ(%)	**LT Scenario**			
	**Without Hidden Nodes**		**With Hidden Nodes**	
	**EDCA/DCF**	**TAROA**	**EDCA/DCF**	**TAROA**
$\left[-10,10\right]$	0.09465±0.00004	0.09394±0.00026	0.05648±0.00117	0.09346±0.00031
$\left[-20,20\right]$	0.09582±0.00002	0.09486±0.00028	0.05645±0.00090	0.09368±0.00045
$\left[-50,50\right]$	0.09383±0.00003	0.09147±0.00018	0.05777±0.00101	0.09150±0.00037
